# Whole-brain white matter organization, intelligence, and educational attainment

**DOI:** 10.1016/j.tine.2019.02.004

**Published:** 2019-06

**Authors:** J. Bathelt, G. Scerif, A.C. Nobre, D.E. Astle

**Affiliations:** a Dutch Autism & ADHD Research Center, University of Amsterdam, NK Amsterdam, The Netherlands; b Department of Experimental Psychology, University of Oxford, United Kingdom; c Oxford Centre for Human Brain Activity, Department of Psychiatry, University of Oxford, United Kingdom; d MRC Cognition and Brain Sciences Unit, University of Cambridge, United Kingdom

**Keywords:** Child development, Connectome, Education, Intelligence, White matter

## Abstract

General cognitive ability, sometimes referred to as intelligence, is associated with educational attainment throughout childhood. Most studies that have explored the neural correlates of intelligence in childhood focus on individual brain regions. This analytical approach is designed to identify restricted sets of voxels that overlap across participants. By contrast, we explored the relationship between white matter connectome organization, intelligence, and education. In both a sample of typically-developing children (*N* = 63) and a sample of struggling learners (*N* = 139), the white matter connectome efficiency was strongly associated with intelligence and educational attainment. Further, intelligence partially mediated the relationship between connectome efficiency and educational attainment. In contrast, a canonical voxel-wise analysis failed to identify any significant relationships. The results emphasize the importance of distributed brain network properties for cognitive or educational ability in childhood. Our findings are interpreted in the context of a developmental theory, which emphasizes the interaction between different subsystems over developmental time.

## 1 Introduction

The ability to reason and solve novel problems is a key human ability, necessary for adapting and learning from a dynamically changing environment. There is a long and rich history of philosophical and scientific exploration of the nature of human intelligence. Indeed, there are many valid ways of defining intelligence. In psychology, intelligence has emerged as a core construct  [13] and understanding individual differences in intelligence has become a central focus across many subfields, including cognitive neuroscience and developmental psychology. In many cases, this has a very practical application - intelligence appears to play a key role in important real-world outcomes, notably school progress  [15]. Intelligence correlates highly with children's educational attainment (0.4–0.7, [43]).

One popular way to measure intelligence is as the shared variance across multiple cognitive tasks that tap different aspects of cognition such as fluid reasoning, executive function, processing speed, short-term and working memory, and spatial reasoning. A highly replicated finding is that performance across tasks loads onto a *general* intelligence factor (*g*) [54] that can be further fractionated into variance associated with specific domains, e.g. verbal vs spatial ability. In this framework, intelligence is conceptualized as a multi-component system that describes individual variation in the ability to reason, solve problems, and think abstractly  [26]. While the relative ranking of individuals on this measure remains remarkably stable over the lifespan  [16], absolute differences are amplified over the course of child and adolescent development  [44]. A strong possibility is that small initial differences in one aspect of intelligence support increases in other aspects  [21], [22]. Accordingly, differences in cognitive ability may contribute to better educational attainment.

Studies on the neural basis of intelligence have shifted from an emphasis on a small number of brain regions to investigations of whole-brain properties. This has in part been driven by new developments in analysis and conceptualization of brain structure and function. Traditional analyses have been focused on detecting local differences that result from brain insults or plasticity and were well aligned with classic localist theories that link such insults or growths/prominences to selective impairments in cognitive function (e.g. [42], [58]). Studies that employed voxel-wise approaches implicated the dorsolateral prefrontal cortex, anterior cingulate cortex, parietal lobe, and medial temporal cortex as the loci of intelligence [19], [35]. In contrast, recent parallel theories in neurocognitive development and network neuroscience have emphasized the role of *interactions among* distributed brain regions [6], [10], [34]. According to this view, cognitive capacity emerges from the contributions of distributed brain regions that function together as an integrated network [5]. Recent advances that capitalize on graph theory, a branch of mathematics concerned with the study of complex systems with interacting elements, indicate that organizational principles of the whole-brain network are strongly linked to general intelligence. In network analysis, the brain is described as a set of nodes, typically brain regions, that are linked through edges that either present white matter connections or statistical associations between brain signals  [50]. A consistent finding is that functional and structural brain networks with higher global efficiency, i.e. networks with shorter connections between any pair of nodes in the network, are associated with higher scores on assessments of general intelligence in both children and adults  [29], [36], [47], [52].

Despite these recent advances in methods for exploring principles of brain organization, these are rarely applied to developmental populations. The vast majority of studies employ methods reliant upon voxel overlap and mass univariate comparisons. This can give the impression that focal differences in brain structure are associated with particular cognitive differences in childhood, and that cognitive difficulties stem from restricted lesion-like effects. However, as noted above, a strong conclusion from developmental theory is that cognitive difficulties that emerge over time are unlikely to be the result of lesion-like effects, but instead should reflect an emergent property of a dynamic system comprised of interacting subcomponents, with difficulties cascading through the system, or being partially compensated for elsewhere. It is difficult to identify these kinds of effects with traditional univariate tests.

The current study explored the relationships between whole-brain white matter network organization, general intelligence, and educational attainment in mid-childhood. We focus on two cohorts of children, one typically developing, recruited from mainstream education, and a second group of struggling learners referred by professionals in children's specialist services. These samples cover a wide range of cognitive performance in development to provide a broad insight into the brain substrates supporting intelligence across average and below-average performance. We focused on white matter because white matter maturation is an important aspect of post-natal brain development with a prolonged trajectory extending into the third decade of life  [39], and it has previously been linked to individual differences in cognition  [9]. Our hypothesis is that graph theoretical measures of global brain organization will be strongly associated with children's general cognitive ability, and that this ability will mediate links between brain organization and educational attainment. Furthermore, we predict that these relationships will not be revealed by a more traditional neuroimaging approach, reliant on voxel overlap across children.

## 2 Participants and methods

### 2.1 Participants

#### 2.1.1 Attention and cognition in education (ACE)

This sample was collected for a study investigating the neural, cognitive, and environmental markers of risk and resilience in children. Children between 7 and 12 years attending mainstream school in the UK, with normal or corrected-to-normal vision or hearing, and no history of brain injury were recruited via local schools and through advertisement in public places (childcare and community centres, libraries). Participating families were invited to the MRC Cognition and Brain Sciences Unit (MRC CBU) for a 2-hour assessment, which included the assessments reported here, and structural MRI scanning. Participants received monetary compensation for taking part in the study. This study was approved by the Psychology Research Ethics Committee at the University of Cambridge (Reference: Pre.2015.11). Parents provided written informed consent and children verbal assent. A total of 89 children participated in the study. Twenty-six children were excluded because of low-quality MRI data (29%, see below for quality control criteria). The final sample consisted of 63 children (34 male, Age: mean = 9.93, std = 1.538, range = 6–12).

#### 2.1.2 Centre for attention, learning, and memory (CALM)

For this study, children aged between 5 and 18 years were recruited on the basis of ongoing problems in attention, learning, language and memory, as identified by professionals working in schools or specialist children's services in the community. Following an initial referral, the CALM staff contacted referrers to discuss the nature of the child's problems. If difficulties in one or more area of attention, learning, language or memory were indicated by the referrer, the family were invited to the CALM clinic at the MRC CBU in Cambridge for a 3-hour assessment. This assessment included the assessments reported here. Exclusion criteria for referrals were significant or severe known problems in vision or hearing that were not corrected or having a native language other than English. Written parental consent was obtained and children provided verbal assent. Families were also invited to participate in MRI scanning on a separate visit. Participation in the MRI part of the study was optional and required separate parental consent and child assent. Contra-indications for MRI were metal implants, claustrophobia, or distress during a practice session with a realistic mock MRI scanner. This study was approved by the local NHS research ethics committee (Reference: 13/EE/0157). Of the full CALM sample, 197 children who participated in neuroimaging and had complete data on all assessments were included for the current analysis. Nine older children at the tail of the age distribution were excluded to focus on a narrower age range. A further 49 participants were excluded because of low-quality neuroimaging data (see below for criteria). The final sample for the analysis consisted of 139 children (90 male, Age: mean = 9.35, std = 1.683, range = 5–13).  The sample included a high proportion of boys as is frequently the case in sample recruited for difficulties in school [51].

### 2.2 Assessments of cognition and educational attainment

#### 2.2.1 Procedure

All children for whom we had cognitive data were tested on a one-to-one basis with a trained researcher in a dedicated child-friendly testing room at the MRC CBU. The battery included a wide range of standardized assessments of cognition and educational attainment. Regular breaks were included throughout the session. Testing was split into two sessions for children who struggled to complete the assessments in one sitting. Measures relating to cognitive performance across different domains were included in this analysis. Tasks that were based on reaction times were not included in this analysis due to their different psychometric properties compared to the included tasks that were based on performance measures.

#### 2.2.2 Fluid reasoning

Fluid intelligence was assessed on the Reasoning task of the Wechsler Abbreviated Scale of Intelligence, 2nd edition [57]. Both children in the CALM and ACE sample completed this assessment.

#### 2.2.3 Working memory

The Digit Recall, Backward Digit Recall, Dot Matrix, and Mr X task of the Automatic Working Memory Assessment (AWMA) [2] were administered individually. In Digit Recall, children repeat sequences of single-digit numbers presented in an audio format. In Backward Digit Recall, children repeat the sequence in backwards order. These tasks were selected to engage verbal short-term and working memory, respectively. For the Dot Matrix task, the child was shown the position of a red dot for 2 s in a series of four by four matrices and had to recall this position by tapping the squares on the computer screen. In the Mr X task, the child retains spatial locations whilst performing interleaved mental rotation decisions. These tasks were selected to engage visual short-term and working memory, respectively. These assessments were the same in the CALM and ACE sample.

#### 2.2.4 Educational attainment

For the ACE sample, tasks from the non-computerized version of the Woodcock-Johnson Test of Achievement, 4th edition (WJ-IV) were administered (*Woodcock-Johnson IV Test of Achievement* 2014).  ‘Letter-Word Identification’ required the reading of letters initially, with the later stages requiring full word reading. ‘Passage Comprehension’ required children to comprehend the semantic context of simple phrases initially (e.g. ‘the cat sat on the mat’, with the children shown a set of pictures), and choose the missing word for longer phrases and passages in later stages of the test. Both assessments employed “discontinue rules” to identify the child's upper limit of ability. A final literacy test was ‘Reading Fluency’. Children were given a set of simple sentences (e.g. ‘the sky is green’) and asked to indicate whether they were true or false. Each child had 3 min to complete as many sentences as possible. Writing abilities were assessed using the ‘Spelling’ subtest. For this test, children were read words and contextual sentences and had to write them down in a booklet. We also included three subtests that measured mathematics ability. The first was ‘Calculation’ and simply required children to perform sums of increasing difficulty. The second measure of mathematics ability was ‘Maths Fluency’. Children were presented with relatively simple calculations in written form and asked to calculate the answers. They were given 3 min to do as many as possible.

For the CALM sample, spelling, reading, and maths measures were taken from the Wechsler Individual Achievement Test (WIAT, [56]). For the spelling assessment, children had to write words starting with simple phonetic words and progressing towards more difficult words with irregular spelling. For the spelling assessment, children were read words along with example sentences and had to write the words in an example booklet. For the reading assessments, children read words starting from short, phonetic words and progressing towards rarer, polysyllabic words. For the math assessment, children had to solve math problems starting with simple retrieval of numeric facts and progressing towards more difficult multi-stage calculations. Correct responses were scored and the assessment was terminated following the rules of the assessment manual.

### 2.3 Magnetic resonance imaging

#### 2.3.1 MRI protocol

Magnetic resonance imaging data were acquired at the MRC CBU, Cambridge U.K. All scans were obtained on the Siemens 3 T Tim Trio system (Siemens Healthcare, Erlangen, Germany), using a 32-channel quadrature head coil. For ACE, the imaging protocol consisted of two sequences: T1-weighted MRI and a diffusion-weighted sequence. For CALM, the imaging protocol included an additional resting-state functional MRI (rs-fMRI) sequence. T1-weighted volume scans were acquired using a whole brain coverage 3D Magnetisation Prepared Rapid Acquisition Gradient Echo (MP-RAGE) sequence acquired using 1 mm isometric image resolution. Echo time was 2.98 ms, and repetition time was 2250 ms. Diffusion scans were acquired using echo-planar diffusion-weighted images with a set of 60 non-collinear directions, using a weighting factor of *b* = 1000 s*mm^−2^, interleaved with a T2-weighted (*b* = 0) volume. Whole brain coverage was obtained with 60 contiguous axial slices and isometric image resolution of 2 mm. Echo time was 90 ms and repetition time was 8400 ms.

#### 2.3.2 MRI quality control

Participant movement may significantly affect the quality of MRI data and may bias statistical comparisons [60]. Several steps were taken to ensure good MRI data quality and minimize potential biases of participant movement. First, children were instructed to lie still and were trained to do so in a realistic mock scanner prior to the actual scan. Second, all T1-weighted images and FA maps were visually inspected by a trained researcher (J.B.) to remove low-quality scans. Further, the quality of the diffusion-weighted imaging (dwi) data were assessed by calculating the displacement between subsequent volumes in the sequence. Scans that showed a displacement of >3 mm within the diffusion-weighted sequence were excluded. Further, we used the maximum displacement across the sequence as a nuisance regressor for all included participants.

### 2.4 White-matter connectome construction

**Fig. 1:**
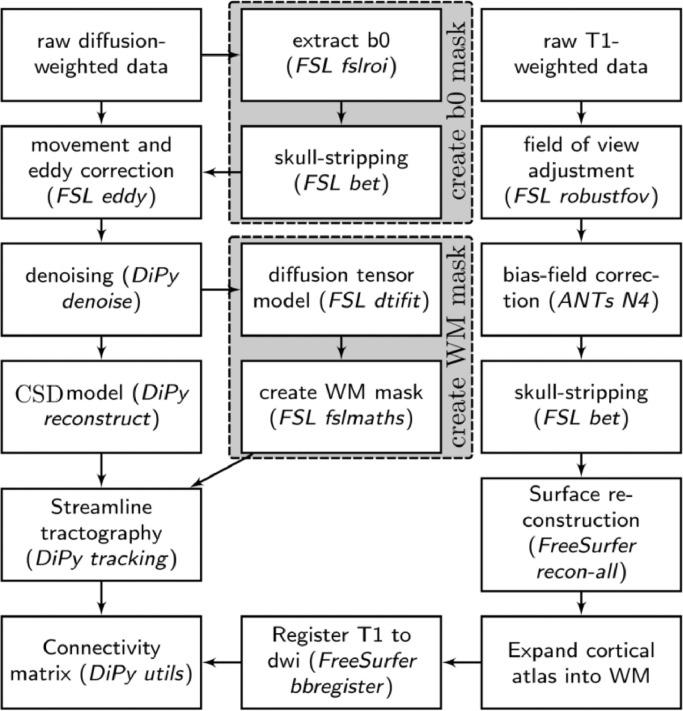
Overview of processing steps to derive the FA-weighted white matter connectome from diffusion- and T1-weighted neuroimaging data.

The white-matter connectome reconstruction followed the general procedure of estimating the most probably white matter connections for each individual, and then obtaining measures of fractional anisotropy (FA) between regions (see [7]). The details of the procedure are described in the following paragraphs (see Fig. 1 for an overview).

For the analysis, MRI scans were converted from the native DICOM to compressed NIfTI-1 format using the dcm2nii tool. Subsequently, a brain mask was derived from the b0-weighted volume of the diffusion-weighted sequence and the entire sequence was submitted for correction for participant movement and eddy current distortions through FSL's eddy tool. Next, non-local means de-noising [11] was applied using the Diffusion Imaging in Python (DiPy) v0.11 package [25] to boost the signal-to-noise ratio. The diffusion tensor model was fitted to the pre-processed images to derive maps of fractional anisotropy (FA) using *dtifit* from the FMRIB Software Library (FSL) v.5.0.6 [8]. A spherical constrained deconvolution (CSD) model [55] was fitted to the 60-gradient-direction diffusion-weighted images using a maximum harmonic order of 8 using DiPy. Next, probabilistic whole-brain tractography was performed based on the CSD model with 8 seeds in any voxel with a General FA value higher than 0.1. The step size was set to 0.5 and the maximum number of crossing fibres per voxel to 2.

For ROI definition, T1-weighted images were preprocessed by adjusting the field of view using FSL's *robustfov*, non-local means denoising in DiPy, deriving a robust brain mask using the brain extraction algorithm of the Advanced Normalization Tools (ANTs) v1.9 [3], and submitting the images to recon-all pipeline in FreeSurfer v5.3 (http://surfer.nmr.mgh.harvard.edu). Regions of interests (ROIs) were based on the Desikan-Killiany parcellation of the MNI template [17] with 34 cortical ROIs per hemisphere and 17 subcortical ROIs (brain stem, and bilateral cerebellum, thalamus, caudate, putamen, pallidum, hippocampus, amygdala, nucleus accumbens). The surface parcellation of the cortex was transformed to a volume using the aparc2aseg tool in FreeSurfer. Further, the cortical parcellation was expanded by 2 mm into the subcortical white matter using in-house software. In order to move the parcellation into diffusion space, a transformation based on the T1-weighted volume and the b0-weighted image of the diffusion sequence was calculated using FreeSurfer's *bbregister* and applied to the volume parcellation. To construct the connectivity matrix, the number of streamlines intersecting both ROIs was estimated and transformed into a density map for each pairwise combination of ROIs. A symmetric intersection was used, i.e. streamlines starting and ending in each ROI were averaged.

### 2.5 Graph theory

The current analysis focused on local and global efficiency (E_j_, E_G_) because this metric has been found to relate closely to measures of intelligence in previous studies [36], [47].  We calculated local and global efficiency for weighted undirected networks as described by Rubinov and Sporns [50]. The shortest path length between two nodes *i* and *j* in a weighted network is defined as $d_{ij}^{w}=\sum a_{uv}\in gi^{w}\Leftrightarrow f\left(w_{uv}\right)$ where *f* is a map from weight to length *and* $g_{ij}^{w}$ is the shortest weighted path between *i* and *j*. The weighted global efficiency (E_G_) is defined as the average of local efficiencies (E_j_): $E_{G}=\frac{1}{n}\sum_{j\in N}\frac{\sum_{j\in N,j\neq i}{\left(d_{ij}^{w}\right)}^{-1}}{n-1}$with *N* as the set of all nodes in the network and *n* the number of nodes.

Spurious connections in streamline tractography are a common problem in structural connectome studies [61]. Typically, a threshold is applied to remove false positive streamlines. However, the choice of this cut-off is largely arbitrary. To remove the effect of setting any particular threshold, a range of density thresholds (0.3 to 0.9) was applied and the area under the curve for each graph metric was compared in subsequent analyses [62].

### 2.6 Statistical analysis

#### 2.6.1 Dimensionality reduction

Exploratory Factor Analysis (EFA) was carried out to reduce the dimensionality of the cognitive assessment and educational attainment data. First, we normalized the raw scores to a mean of 0 and unit standard deviation (z-score) and checked the normed data for univariate (>3 standard deviations above the mean) and multivariate outliers (Mahalanobis distance). No outliers were detected for the cognitive or educational attainment data in the ACE nor the CALM sample. Next, we calculated bivariate Pearson correlations between the cognitive and educational attainment scores and carried out exploratory factor analysis (EFA) using the *psych* v1.7.8 package under *R* 3.4.3 using the maximum likelihood implementation. The number of factors was estimated via parallel analysis that compared the scree plot of the observed data to a bootstrapped sample of 10,0000 permutations [24]. The optimal solution was indicated as the highest number of factors that had an eigenvalue above the eigenvalue obtained for scrambled data. The factor score was calculated for this solution. Subsequently, we regressed the linear and quadratic effects of age and the effect of gender from the factor scores.

#### 2.6.2 Regression analysis

Regression analyses were carried out using the scientific python (SciPy) v0.18.1 and *statsmodels* v0.6.1 packages for Python. Visualizations were created using the *matplotlib* v1.5.1 package for Python. The mediation analyses were performed using the *lavaan* package v0.5 in R [49].

### 2.7 Comparison analysis with tract-based spatial statistics

To contrast the structural connectome approach with more commonly used voxelwise statistical analysis, FA maps were processed using tract-based spatial statistics (TBSS) as implemented in FSL v5.0.9. (see [53] for detailed description of TBSS). In short, FA maps were moved to common space via affine and non-linear transformations using FSL tools. A common template based on a large developmental sample constructed using advanced normalization tools (ANTs) v1.9 [4] was used as the registration target (NKI Rockland Enhanced Sample, [45]). Next, the mean FA image was created and thinned to create a mean FA skeleton which represents the centers of all tracts common to the group. Each subject's aligned FA data was then projected onto this skeleton.

For statistical comparison, the positive and negative association between FA values and cognitive or educational attainment factor scores was evaluated controlling for the effect of age, sex, movement, and intracranial volume in a generalized linear model (GLM). The model also contained an intercept term. Inflation of error rates due to multiple comparison across voxels was controlled using cluster-free threshold enhancement as implemented in FSL randomise  [59].

## 3 Results

### 3.1 Factor analysis of cognitive and educational attainment measures

**Fig. 2:**
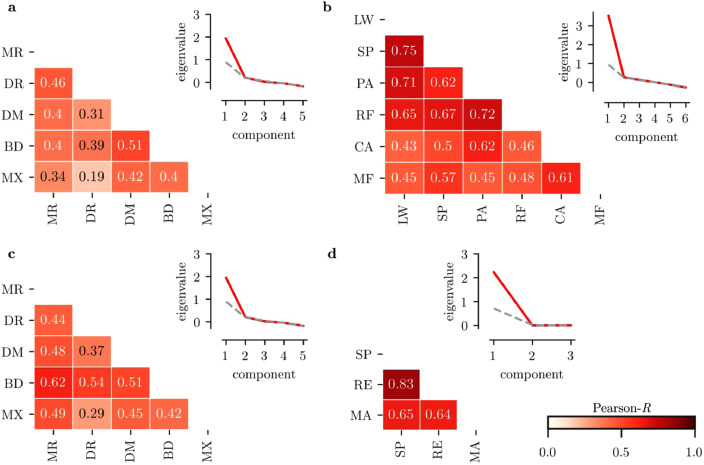
Exploratory factor analysis (EFA) of cognitive (a,c) and educational attainment measures (b,d) in the ACE (a,b) and CALM sample (c,d). The left panel of each plot shows a matrix of correlations between measures for each factor. The right panel shows the eigenvalues for each principal component (red) compared to scrambled data (grey). Abbreviations: MR=matrix reasoning, DR = Digit Recall, DM = Dot Matrix, BD = Backward Digit Recall, MX = Mr. X, SP = Spelling, RE = Reading, MA = Maths.

We applied exploratory factor analysis (EFA) to investigate the factor structure of cognitive and educational attainment assessments in a maximum likelihood procedure. Parallel analysis that compared the original data to a bootstrap sample of 10,000 random permutations favoured a one-factor solution for the cognitive and the attainment measures in both samples (see scree plots in Fig. 2). Fit indices indicated that the one-factor model provided a good account of the cognitive and educational attainment data (RMSR < 0.08, RMSEA < 0.2, Tucker-Lewis index > 0.95, seeTable 3) with the exception of the educational assessment data in the ACE sample where the Tucker-Lewis index was below the recommended cut-off. A solution with two factors only marginally improved the Tucker-Lewis index (0.834 for 2 factors compared to 0.826 for 1 factor) and lead to worse root-mean-squared error indices (RMSR: 0.08, RMSEA: 0.202). The correlation matrix (see Fig. 2) suggested that the educational assessments in the ACE sample could represent two factors related to reading/spelling and maths respectively. However, with only two assessments, the arithmetic factor may not have been adequately represented to fully capture the unique variance associated with maths performance. Given that parallel analysis and the other fit indices suggested a one-factor solution for the educational attainment data in the ACE sample, we opted to retain this solution for further analysis.

**Table 1:** Scores normalized according to the mean and standard deviation of the normative sample. The statistics tested if the observed values differed from the age-expected values with a mean of 0.

Sample	Measure	mean	std	*t*	*p*
CALM	Matrix reasoning	−0.63	0.938	−7.93	<0.001
	Digit recall	−0.49	1.157	−5.04	<0.001
	Dot matrix	−0.49	0.956	−6.02	<0.001
	Backward digit recall	−0.6	0.792	−8.87	<0.001
	Mr X	−0.22	0.924	−2.8	0.006
ACE	Matrix reasoning	0	0.89	0.03	0.978
	Digit recall	0.31	0.795	3.12	0.003
	Dot matrix	0.42	0.936	3.58	0.001
	Backward digit recall	0.29	0.986	2.34	0.022
	Mr X	0.47	1.051	3.56	0.001
CALM	Spelling	−1.17	0.886	−15.57	<0.001
	Reading	−0.96	1.178	−9.64	<0.001
	Maths	−1.05	1.141	−10.89	<0.001
ACE	Letter-Word identification	0.66	0.801	6.52	<0.001
	Spelling	0.5	1.037	3.84	<0.001
	Passage comprehension	−0.1	0.78	−0.99	0.326
	Reading fluency	0.29	1.065	2.14	0.036
	Calculation	0.26	1.088	1.86	0.067
	Math fluency	−0.39	0.853	−3.61	0.001

**Table 2:** Results of simple regression between outcome variables and nuisance variables. Significant effects at *p* < 0.05 are highlighted in bold print.

		cogn.		ed.		E_G_	
		*β*	*p*	*β*	*p*	*β*	*p*
ACE	Age	**−0.2**	**0.031**	**−0.3**	**0.009**	−0.1	0.496
	Age^2^	**0.26**	**0.016**	**0.29**	**0.007**	**−0.2**	**0.032**
	Brain vol.	0.07	0.542	0.13	0.228	0.14	0.216
	Sex	−0.1	0.819	−0.1	0.653	**0.86**	**<0.001**
	Motion	**0.22**	**0.041**	0.05	0.645	**−0.2**	**0.028**
CALM	Age	**0.57**	**<0.001**	**0.62**	**<0.001**	**0.2**	**0.017**
	Age^2^	0.01	0.882	0.04	0.527	−0	0.81
	Brain vol.	0.02	0.856	−0.2	0.059	**0.33**	**<0.001**
	Sex	0.21	0.251	0.01	0.967	**0.57**	**0.001**
	Motion	−0.1	0.2	−0.1	0.154	−0.1	0.504

**Table 3:** Results of the Exploratory factor analysis (EFA) of cognitive and educational assessment data in the ACE and CALM sample. The table shows the standardized loading of each scale on a common factor. The confidence interval was based on a bootstrap sample with 10,000 permutations. Fit indices for each solution are shown: var explained = variance explained, RMSR: standardized root-mean squared residual, RMSEA: root-mean squared error of approximation, Tucker-Lewis = Tucker-Lewis normed fit index.

**ACE**					
	Loading	5%	95%	Fit indices	
Matrix reasoning	0.62	0.42	0.85	var explained	0.39
Digit recall	0.53	0.29	0.8	RMSR	0.06
Dot matrix	0.69	0.48	0.88	RMSEA	0.058
Backward digit recall	0.71	0.53	0.87	Tucker-Lewis	0.973
Mr X	0.55	0.34	0.77		

Letter-Word	0.83	0.73	0.92	var explained	0.71
Spelling	0.83	0.72	0.93	RMSR	0.04
Passage comprehension	0.83	0.73	0.94	RMSEA	0.198
Reading fluency	0.81	0.71	0.9	Tucker-Lewis	0.834
Calculation	0.64	0.45	0.8		
Math Fluency	0.62	0.43	0.79		

**CALM**					
	Loading	5%	95%		
Matrix reasoning	0.75	0.65	0.85	var explained	0.48
Digit recall	0.62	0.49	0.75	RMSR	0.04
Dot matrix	0.66	0.54	0.78	RMSEA	0.059
Backward digit recall	0.82	0.73	0.91	Tucker-Lewis	0.979
Mr X	0.59	0.46	0.72		

Spelling	0.93	0.86	0.98	var explained	0.74
Reading	0.91	0.85	0.96	RMSR	<0.01
Maths	0.74	0.65	0.83	RMSEA	<0.001
				Tucker-Lewis	>0.999

Regarding the factor loading, Dot Matrix and Backward Digit Recall had the highest loading on the cognitive factor in the ACE sample, while Matrix Reasoning and Backward Digit Recall had the highest loading in the CALM sample (see Table 3). However, all cognitive assessment showed a high loading on the cognitive factor across both samples. For the educational attainment factor, reading and spelling measures showed a higher loading on the educational factor across both samples in line with their greater representation in the assessment tasks compared to the maths assessments (see Table 3).

### 3.2 Relationship between global efficiency, cognitive factor scores, and educational attainment scores

**Fig. 3:**
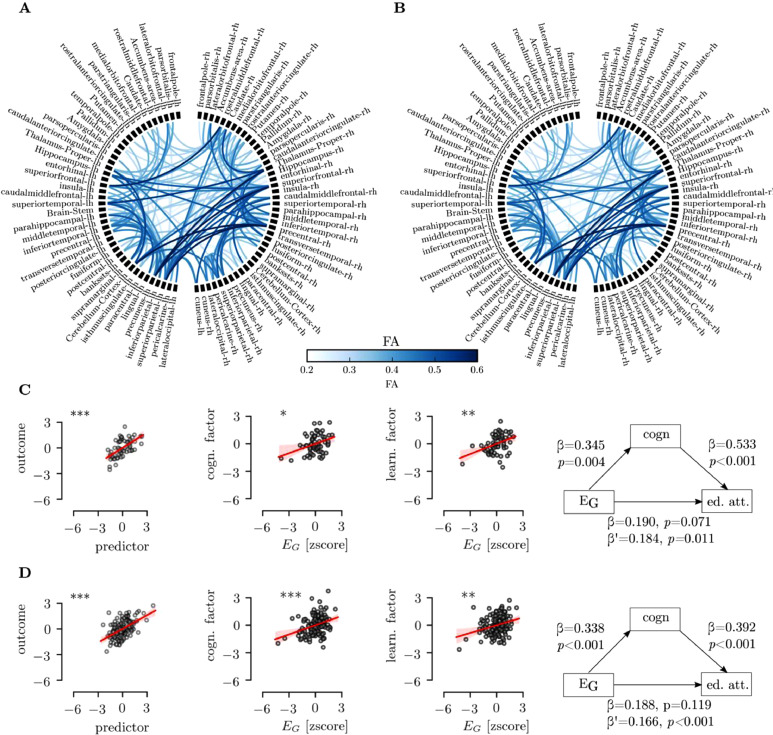
(A) Visualization of the group-average connectome in the ACE sample (B) Group-average connectome in the CALM sample. (C) Regression of factor scores for educational attainment, cognition, and global efficiency of the white matter network and mediation relationships for the ACE sample. The shaded area in the regression results shows the 5–95%ile confidence interval based on bootstrap sample with 500 permutations. (D) Regression and mediation results in the CALM sample. Legend: *β*: direct effect,  *β*’: indirect effect,  ***p<0.001, **p<0.01, *p<0.05.

Regression analysis indicated that cognitive factor scores were a strong predictor of educational attainment factor scores (ACE: F(1,61) = 35.24, *R*^2^ = 0.37, *β* = 0.61, *p* < 0.001; CALM: F(1,137) = 75.75, *R*^2^=0.36, *β* = 0.61, *p*<0.001) that explained over 35% of variance in educational attainment scores. E_G_ was also strongly related to cognitive factor scores in the ACE and CALM sample (ACE: F(1,61) = 8.23, *R*^2^ = 0.119, *β* = 0.345,  *p* = 0.006; CALM: F(1,137) = 17.61, *R*^2^ = 0.114, *β* = 0.338, *p* < 0.001). Further, E_G_ was also related to educational attainment scores in both sample (ACE: F(1,61) = 9.914, *R*^2^ = 0.141, *β* = 0.374, *p* = 0.003; CALM: F(1,137) = 12.02, *R*^2^ = 0.08, *β* = 0.284, *p* = 0.001). Next, we investigated the relationships between all variables in a mediation model. The results indicated that cognitive factor scores were mediating the relationship between E_G_ and educational attainment scores in both samples (see Fig. 3). The results indicated that the relationship between E_G_ and learning was partially mediated by indirect effect via cognition.

### 3.3 Regional associations

**Fig. 4:**
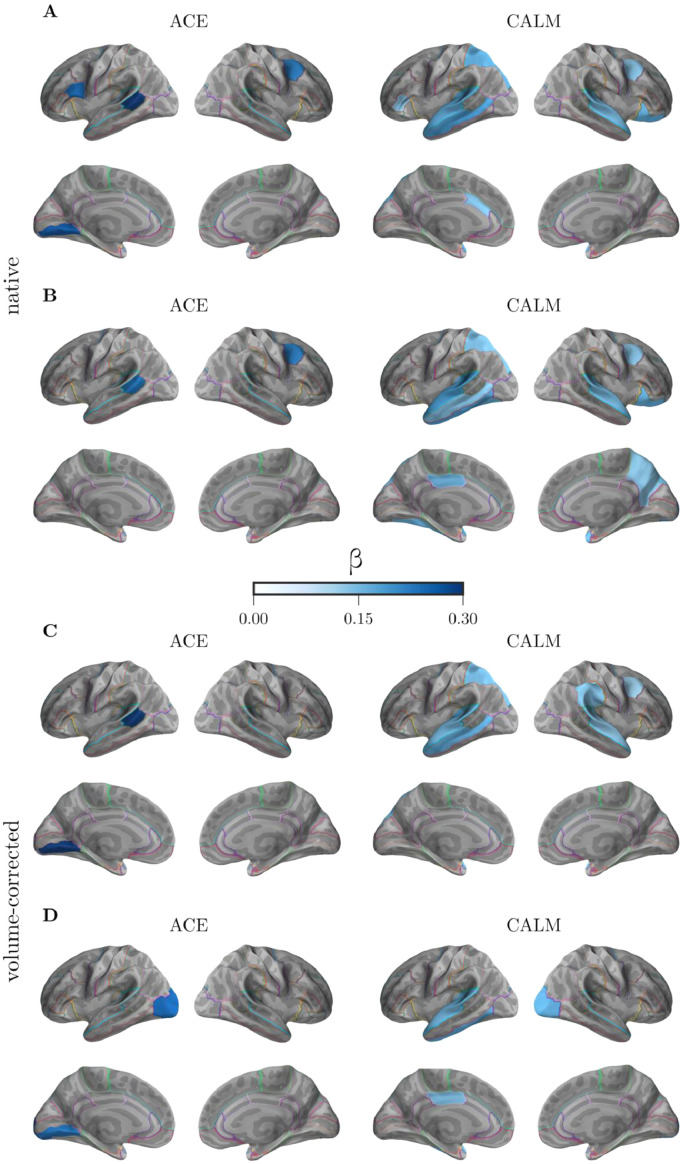
Regression coefficients for the regional associations between local efficiency (E_j_) and the cognitive factor scores (A, C) and the learning factor scores (B, D) in the ACE and CALM sample. The top panel shows native associations and the bottom panel shows associations corrected for the grey matter volume of each region.

To identify brain regions that were most closely associated with cognitive and educational attainment outcomes, we assessed the strength of the linear association between local efficiency (E_j_) and factor scores in the ACE and CALM sample (see Fig. 4). For cognitive factor scores, the association with E_j_ was strongest for the left banks of the superior temporal sulcus (*β* = 0.24), left medial occipital cortex (*β* = 0.23), and right caudal middle frontal cortex (*β* = 0.23) in the ACE sample, and with the left middle temporal (*β* = 0.21), transverse temporal (*β* = 0.18), superior temporal (*β* = 0.17), superior parietal (*β* = 0.17), and anterior cingulate cortex (*β* = 0.14), and the right lateral orbitofrontal (*β* = 0.17), superior temporal (*β* = 0.15), caudal middle frontal cortex (*β* = 0.14) in the CALM sample (Fig. 4A). For educational attainment scores, the association with E_j_ was strongest for the left banks of the superior temporal sulcus (*β* = 0.33), lingual gyrus (*β* = 0.27), and middle frontal gyrus (*β* = 0.25) in the ACE sample, and for the left lateral occipital (*β* = 0.21), superior temporal (*β* = 0.19), middle temporal (*β* = 0.14), inferior temporal (*β* = 0.19), transverse temporal (*ββ* = 0.15), fusiform (*β* = 0.17), and superior parietal cortex (*β* = 0.15), and the right temporal pole (*β* = 0.16) and superior parietal cortex (*β* = 0.15) in the CALM sample (see Fig. 4B). Since larger brain region have a higher chance for intersections with white matter streamlines which may confound the results ([27], [30], [32]), we included the grey matter volume of regions as a control variable in an additional analysis. For the ACE sample, the association between the cognitive factor and the left banks of the superior temporal sulcus and left medical occipital sulcus remained after this correction (see Fig. 4C), while the association with the right caudal middle frontal cortex was attenuated. The association with the learning factor changed in the ACE sample when volume differences between regions were corrected (Fig. 4D). With volume correction, the learning factor was associated with the left medial and lateral occipital cortex. The CALM sample showed a similar pattern of results. The association between temporal and parietal regions with the cognitive factor was still observed when correcting for grey matter volume, while associations with middle frontal areas (left caudal middle frontal, right lateral orbitofrontal cortex) disappeared (Fig. 4C). Further, the associations between the learning factor and E_j_ in the CALM sample were sparser but showed similar involvement of the left temporal areas and the left medial cingulate cortex. Similar to the ACE results, an additional involvement of the lateral occipital cortex was indicated when correcting for grey matter volume in the CALM sample (Fig. 4D).

### 3.4 Voxel-wise associations with cognition and educational attainment

**Fig. 5:**
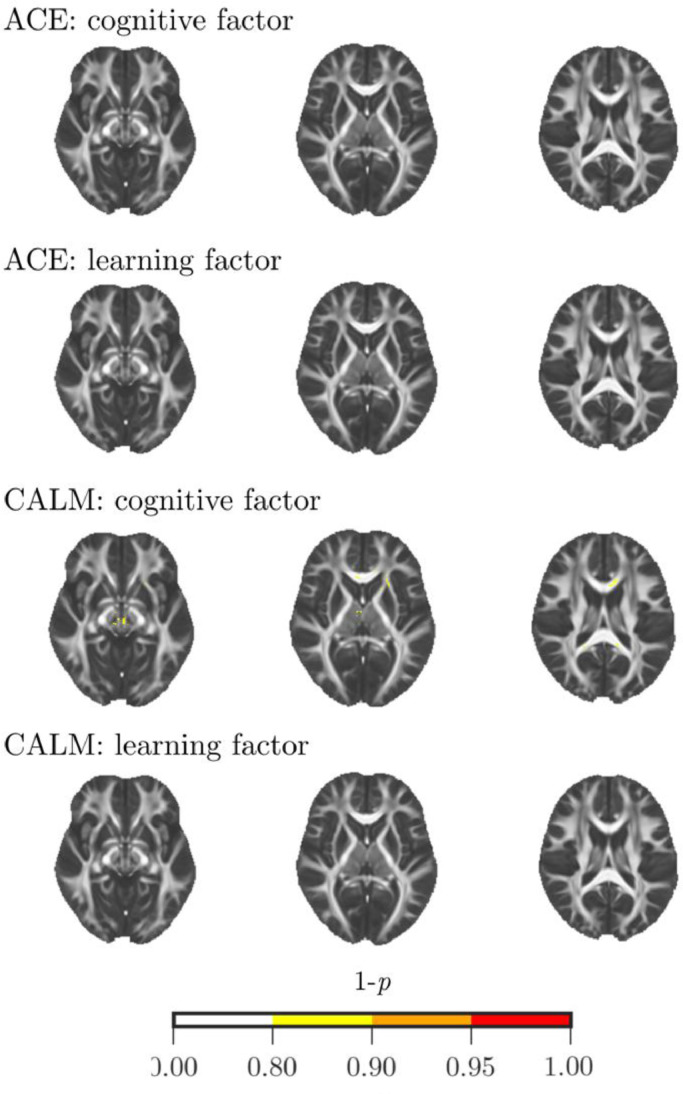
Results of voxel-wise analysis using tract-based spatial statistics (TBSS). There were no significant positive or negative associations between voxel FA values and cognitive or educational attainment measures in the ACE or CALM sample (*p*_corrected_ > 0.1). Only maps of positive associations are shown.

We performed an alternative analysis to evaluate the association between voxel-wise FA and cognitive and educational attainment scores. There were no statistically significant positive or negative associations at *p*_corrected_ < 0.05 nor at *p*_corrected_ < 0.1 (see Fig. 5).

## 4 Discussion

The current study investigated the relationship between cognitive ability, educational attainment, and the global and local efficiency of the white matter connectome. Global efficiency of the white matter connectome was strongly associated with children's cognitive and educational ability - higher global efficiency was related to better general intelligence, both in a sample of children with age-expected ability and a sample of struggling learners.

In a first step, we derived factors for cognition and learning in our two independent samples of typically-developing children and struggling learners. For cognitive measures, a single-factor solution was favoured for both samples that explained between 40 and 50% of the variance in cognitive scores and loaded roughly equally on measures of fluid reasoning and visuospatial and verbal short-term and working memory. This factor is likely to reflect *general* intelligence (*g*) given the similarity of the current results with meta-analytic studies of this construct  [33]. However, four of the five measures used to derive the cognitive factor were short-term and working memory tasks. Intelligence and working memory are separate constructs that can be distinguished using factor analysis ([1], [20]), although typically using factor rotation or confirmatory factor analysis to tease the factors apart and with high correlations between the factor (0.85 according to Oberauer et al. [46]). The single-factor solution in the current study may be skewed towards a higher contribution of working memory and may only partially reflect contributions of a separate fluid intelligence construct. However, general intelligence may also be regarded as a hierarchical structure with an overarching shared variance between all cognitive measures that can be subdivided into separate domains, including short-term and working memory. This may explain the roughly equal contributions of Matrix Reasoning and the short-term and working memory tasks for the first factor in the current analysis. The current study also finds a high correlation between academic attainment tasks aimed at assessing reading, writing, and arithmetic that were consistently observed in two independent samples and using different standardised assessments. Factor analysis also favoured a single factor solution for the learning assessments. Reading and maths have generally been regarded as separate domains with specific mechanisms being associated with learning in each domain. This is also reflected in categorisation of learning difficulties that emphasizes specific deficits is associated with reading (dyslexia) and maths (dyscalculia). However, recent studies show a high degree of overlap that indicates that performance in one domain is highly related to performance in another [37]. Higher correlations within domains (reading/writing vs maths, especially in the ACE sample) indicate that the domains may be separable, e.g. through factor rotation, but the high correlation across all tasks suggests an overriding single factor that explains a large degree of variance. The single factor for learning may reflect similarities in the assessment, i.e. standardised assessment with similar materials and instructions by the same assessor, or may reflect a common source of variance that has a similar impact on academic attainment. This common source of variance may stem from a common constraint through general intelligence as suggested by the close relationship between the cognitive and learning factors in the current analysis. In both samples, cognitive factor scores explained more than 35% of the variance in the learning factor. This finding fits in well with meta-analyses showing that fluid reasoning is closely related to school performance  [38], [41], particularly when structured assessments are used to assess academic attainment  [18]. In a second step, we assessed the relationship between global efficiency (E_G_) of the white matter network with general intelligence and educational attainment factor scores. The results indicated that E_G_ was related to general intelligence replicating findings in adults and children  [28], [36], [40]. This relationship was observed in a sample of children with scores within the typical range and a sample of struggling learners, which suggests that the relationship between global white matter organisation and general intelligence extends to the lower performance range. The analysis of regional associations showed different patterns for the CALM and the ACE sample. In the CALM sample, white matter connections of frontal, parietal, and temporal regions were most strongly associated with cognition and educational attainment, even when correcting for the effect of grey matter volume. These associations agree with previous studies in adults that reported associations between intelligence and structural imaging markers in the prefrontal cortex, parietal lobe, and medial temporal cortex [14], [35]. One possibility is that these regions play a key role in integrating whole-brain neuronal activity, acting as hubs [31]. Regional associations in the ACE sample were sparse in comparison showing only associations with cognition and educational attainment in the superior temporal lobe, frontal gyrus, and occipital regions. Part of the sparsity may be attributable to the lower statistical power in the ACE sample due to the lower sample size. Alternatively, domain-specific processing, i.e. processing within the language system (frontal and temporal) and visual system (medial and lateral occipital), may constrain performance in the ACE compared to the CALM sample that displays a greater reliance on association areas (prefrontal and parietal associations). Such a shift from domain-general to domain-specific processing has been suggested across development [34] and may reflect performance differences between the groups. The regional associations notwithstanding, the graph analytic results suggest that properties of the whole-brain white matter connectome are more closely related to general intelligence and educational attainment than white matter integrity of any particular white matter substrate. Inferior connectivity in any part of the network may be compensated for by better connectivity elsewhere [23], which may explain the importance of whole-brain properties and lack of overlap across individuals in the voxel-wise TBSS analysis.

It is important to bear in mind that the results of the current study come with some limitations. First, we used samples of children with typical performance and children referred for difficulties in school. It is not clear from the current analysis if associations between white matter network properties, intelligence, and educational attainment extend to superior performance. In addition, the analysis was based on pre-collected data with assessment protocols that were not optimised to assess general intelligence. Better coverage of different aspects of cognition and learning, e.g. additional assessments of maths and non-verbal reasoning, would have provided a more adequate characterisation that would have better reflected general ability across domains. Further, both samples were cross-sectional, which precludes the analysis of age-related associations. Longitudinal data would allow us to explore how changes in structural connectomics are linked with improvements in educational attainment and cognitive ability. Another potential caveat concerns the methodology of the connectome construction in the current study. A multitude of methods for constructing structural connectomes from diffusion-weighted data have been proposed with little validation of methods through histological comparisons [48]. The methods employed in the current study were chosen to reflect recommended practices [12], but their relationship to histological measurements remains to be validated.

In conclusion, the results of the current analysis indicate that higher global efficiency of the white matter connectome is associated with better general intelligence and educational attainment in children and adolescents with performance in the age-expected and below age-expected range. These findings support views that emphasize the importance of distributed networks for higher-level cognitive processes ([34], [5].

## Conflict of interest statement

The authors whose names are listed immediately below certify that they have NO affiliations with or involvement in any organization or entity with any financial interest (such as honoraria; educational grants; participation in speakers’ bureaus; membership, employment, consultancies, stock ownership, or other equity interest; and expert testimony or patent-licensing arrangements), or non-financial interest (such as personal or professional relationships, affiliations, knowledge or beliefs) in the subject matter or materials discussed in this manuscript.

## Ethics statement

Study 1 “Attention and Cognition in Education (ACE)”: This study was approved by the Psychology Research Ethics Committee at the University of Cambridge (Reference: Pre.2015.11). Parents provided written informed consent and children verbal assent.

Study 2 “Centre for Attention, Learning, and Memory (CALM)”: This study was approved by the local NHS research ethics committee (Reference: 13/EE/0157). Parents provided written informed consent and children verbal assent for their participation in the cognitive assessment part of the study, and separate written informed consent and verbal assent for participation in the brain imaging part of the study.

## Financial disclosure

This work has been supported by Medical Research Council intramural programmes (MC-A0606-5PQ41 to J.B. and D.A.). The Centre for Attention Learning and Memory (CALM) research clinic is based at and supported by funding from the MRC Cognition and Brain Sciences Unit, University of Cambridge.

G.S. was supported by a James S. McDonnell Understanding Human Cognition Award. K.N. has been supported by a Wellcome Trust Senior Investigator Award (ACN 104571/Z/14/Z) and by the NIHR Oxford Health Biomedical Research Centre. The Wellcome Centre for Integrative Neuroimaging is supported by core funding from the Wellcome Trust (203139/Z/16/Z).

## Appendix Supplementary materials

Image, application 1
